# Air Pollution and Dispensed Medications for Asthma, and Possible Effect Modifiers Related to Mental Health and Socio-Economy: A Longitudinal Cohort Study of Swedish Children and Adolescents

**DOI:** 10.3390/ijerph14111392

**Published:** 2017-11-16

**Authors:** Anna Oudin, Lennart Bråbäck, Daniel Oudin Åström, Bertil Forsberg

**Affiliations:** Occupational and Environmental Medicine, Umeå University, 90187 Umeå, Sweden; Lennart.Braback@umu.se (L.B.); Daniel.oudin_astrom@med.lu.se (D.O.Å.); bertil.forsberg@umu.se (B.F.)

**Keywords:** asthma, childhood asthma, air pollution, stress, socio-economy, mental health

## Abstract

It has been suggested that children that are exposed to a stressful environment at home have an increased susceptibility for air pollution-related asthma. The aim here was to investigate the association between air pollution exposure and asthma, and effect modification by mental health and by socio-economic status (as markers of a stressful environment). All individuals under 18 years of age in four Swedish counties during 2007 to 2010 (1.2 million people) were included. The outcome was defined as dispensing at least two asthma medications during follow up. We linked data on NO_2_ from an empirical land use regression to data from national registers on outcome and potential confounders. Data was analyzed with logistic regression. There was an odds ratio (OR) of 1.02 (95% Confidence Interval (CI: 1.01–1.03) for asthma associated with a 10 µg·m^−3^ increase in NO_2_. The association only seemed to be present in areas where NO_2_ was higher than 15 µg·m^−3^ with an OR of 1.09 (95% CI: 1.07–1.12), and the association seemed stronger in children with parents with a high education, OR = 1.05 (95% CI: 1.02–1.09) and OR = 1.04 (95% CI: 1.01–1.07) in children to mothers and father with a high education, respectively. The association did not seem to depend on medication history of psychiatric disorders. There was weak evidence for the association between air pollution and asthma to be stronger in neighborhoods with higher education levels. In conclusion, air pollution was associated with dispensed asthma medications, especially in areas with comparatively higher levels of air pollution, and in children to parents with high education. We did not observe support for our hypothesis that stressors linked to socio-economy or mental health problems would increase susceptibility to the effects of air pollution on the development of asthma.

## 1. Introduction

Asthma is one of the most common chronic diseases in children. Despite decades of intense research, the etiology and pathogenesis are still partly unknown, although environmental exposures in infancy [1] and stress are of importance [1,2,3,4,5,6,7]. Negative life events can cause asthma attacks in children, especially if they are exposed to chronic stress [8]. Furthermore, there is a strong association between respiratory symptoms and psychological status [9], and parents’ mental health is associated with childhood asthma in the offspring [10]. The causal relationship between asthma and mental health/stress seem to point in both directions because asthma is strongly related to perceived life quality but can also yield anxiety and fatigue. Childhood asthma has received a lot of attention as a cause for mental health problems as it, for example, is a risk factor for neuropsychiatric disease [11].

Long-term exposure to air pollution seem to be a risk factor for asthma [2,12], reduced lung function [13], and sensitization [14], although some large studies have been negative [15]. Interestingly, it has been suggested that children that are exposed to a stressful environment at home have an increased susceptibility for air pollution-related asthma [16,17].

Physical health and mental health are thus intertwined, but air pollution can also have a direct negative effect on the brain, for example cognitive development [18,19], cognitive decline and dementia [20,21,22]. Experimental studies show that an association between air pollution exposure and mental health is plausible [23]. Anxiety disorders and schizophrenia are more common in urban areas [24,25], which can be attributed to urban social environment, but possibly thus also to environmental factors. We recently showed that air pollution was associated with medications for psychiatric disorders in Swedish children and adolescents [26], and there is support for air pollution exposure during fetal life to increase risk of autism [27,28], and for air pollution to be linked to behavioral problems in children [29,30].

**Figure 1:**
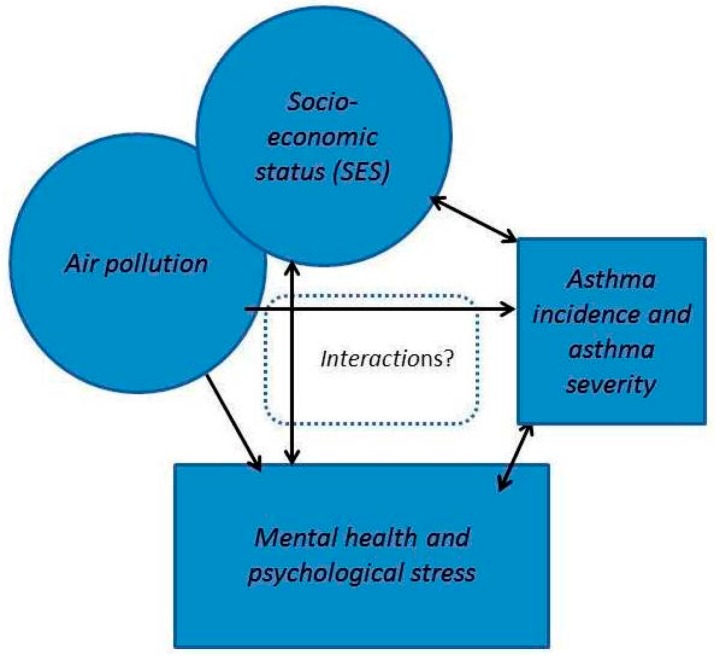
Possible causal pathways between exposure to air pollution, mental health, socioeconomic status and asthma.

In summary, the relationship between asthma, mental health, stress, socio-economy and air pollution is likely highly complex (Figure 1). Our hypothesis is that mental health and socio-economy modify the association between air pollution and pediatric asthma, with the theory that stressors linked to socio-economy or mental health problems directly lead to deteriorated health, that susceptibility for air pollution is increased through alterations in the immune system or other biological systems [31], that city dwellers react differently to stress than persons residing in rural areas [32], or that air pollution cause affective responses or impair cognition.

The aim of the present study was to investigate the association between air pollution exposure and dispensed medications for asthma, and if that association was modified by mental health or by socio-economic status, in a large cohort of Swedish children and adolescents, to somewhat disentangle this complex relationship.

## 2. Materials and Methods

### 2.1. Study Area and Cohort

**Figure 2:**
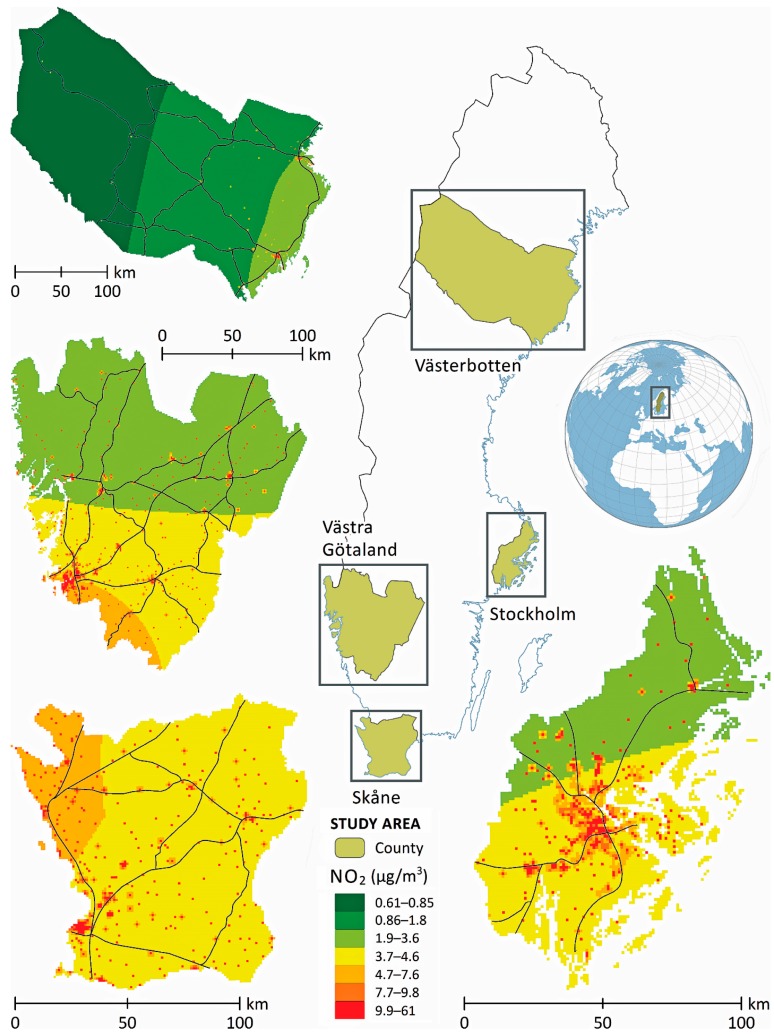
Concentrations of NO_2_ modelled by the Swedish Research Institute in the four counties of the study area.

The study area and cohort have been described in detail elsewhere [26], but briefly, we used a prospective cohort design where all individuals under 18 years of age who at any time during 1 January 2007–31 December 2010 had a registered residential address in any of the four Swedish counties of Stockholm, Västra Götaland, Skåne and Västerbotten (Figure 2).

The four counties encompass more than half the Swedish population and are heterogeneous in terms of geographical location, population size and population density but also with respect to migration, socioeconomic characteristics, and urbanization and air pollution concentrations.

### 2.2. National Register Data

We used data from the Umeå SIMSAM lab [33]. Data in the lab comes from national registers (which covers the entire Swedish population) and local registers, which were combined via the personal identification number for the use of research. For the present study, we used data from the Swedish National Board of Health and Welfare on a group of dispensed medication related to asthma, both long acting and rescue medications, namely medications starting with the following Swedish ATC codes: R03AC (selective beta-2 stimulating medications), R03AK (adrenergic and other medications for obstructive airway disease), R03BA (glucocorticoids), R03BC (anti allergic medications, except corticosteroids), R03CC (selective beta-2-stimulating medications) and R03DC (leukotriene receptor antagonists). We defined the outcome as dispensing at least two dispensed medications with any of these codes. By defining the outcome as at least two dispensed asthma medications during follow-up, our aim was to capture patients more likely to have asthma than patients who only dispensed asthma medications once. Many children with respiratory symptoms, especially very young children, are prescribed asthma medications to evaluate if their symptoms decrease by medication. We thus wanted to avoid to classify children with unspecified respiratory symptoms as asthmatics. We also used data on dispensed medications for psychiatric disorders, namely medications with an ATC-code starting with N05 and N06, hereafter referred to as N05 and N06. N05 consists of neuroleptics (antipsychotic medications), ataractics and sleeping pills (a broad group of sedative medications including hydroxyzine and melatonin-based medications). N06 consist mainly of anti-depressants and ADHD medications when prescribed to children. We defined two variables as dispensed N05 or N06 during baseline (when follow-up started). From the medical birth registry we used information on maternal body mass index in early pregnancy (continuous variable) and maternal smoking during early pregnancy (three categories) Furthermore, from Statistics Sweden we used data on age at the start of follow-up (continuous variable), sex, maternal and paternal education level (four categories) at start follow-up and yearly data on parental unemployment (yes/no). Based on data from Statistics Sweden we defined a group-level (neighborhood) variable on socioeconomic status on Small Areas for Market Statistics (SAMS), namely the proportion of the population in the SAMS area with three or more years of undergraduate studies in the age category 25–65 years. SAMS are supposed to represent homogeneous neighborhoods and there are 6016 SAMS areas in our study area. The quartiles of the variable was <14%, 14% < 20%, 20% < 32% and ≥32% with three or more years of undergraduate studies in the age category 25–65 years.

### 2.3. Air Pollution Exposure Assessment

The Swedish environmental research institute has developed an empirical Land Use Regression model to estimate the urban contribution of NO_2_ added to the regional background level. The model is based on the ratio of the urban content contribution, the meteorological parameters and the population distribution. It takes into account that the levels are not evenly distributed across a city but related to population density by including the spatial distribution of the urban contribution [34]. The base year of the land use regression model was 2010 and the spatial resolution was 1 km^2^. The model has previously been showed to have fairly good accordance with a dispersion mode [35].

This model was used together with a model for the regional background levels built on monitoring data to calculate exposure estimates of NO_2_ the entire Swedish population, for the year when the individual was included in the study (any year between 2005 and 2010). We used this measure as a marker of long-term exposure to air pollution with the underlying assumptions that the concentration at study inclusion was a valid marker for long-term exposure, and that the spatial contrasts in exposure during follow-up were fairly constant. The study was approved by the regional ethics board in Umeå (Dnr 2010-157-31).

### 2.4. Statistical Analysis

**Figure 3:**
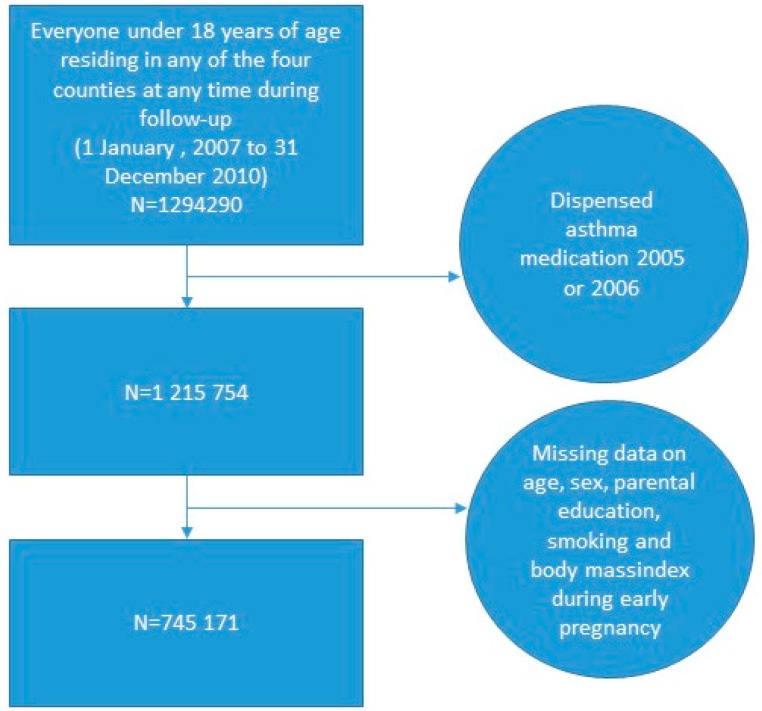
Study flow-chart.

The data was analyzed with logistic regression. The results are presented as Odds ratios (ORs) and their 95% CIs. We studied NO_2_ as a continuous variables, and present ORs associated with pollutant increases of 10 μgm·^−3^. The initial cohort size was 1,294,290, but we excluded study persons with any dispensed asthma medication during 2005 or 2006 to somewhat capturing incident cases, and the cohort size was reduced to 1,215,754 individuals (Figure 3). It should be noted that we had no records of dispensed medications before 2005, and therefore children denoted incident cases in our study may be not be true new cases, but only new cases since 2005. Individuals with missing data on any of the variables included in the main adjusted model (age, sex, parental education, smoking and BMI during early pregnancy) were excluded from the main analysis. The total size of the complete cohort was 745,171 individuals. We used an open cohort approach. Individuals who moved into, or out of, the study areas during follow-up were thus included or excluded in the cohort on December 31 (due to register-technicalities) of the year they moved in or out. Children who were born or died during follow-up were included/excluded at time of birth/death.

In an additional analysis, we adjusted the models for maternal and paternal income. Further, we stratified our analyses on parental education, previous dispensed N05 or N06, parental unemployment, and area SES. We also ran age-specific analyses for the age at baseline groups 0 < 5, 5 < 10, 10 < 15 and 15–18. We evaluated the presence of heterogeneity in the county-specific results by including a cross-product term in the equation. Otherwise we refrained from calculating *p*-values for effect modification since the size of the cohort would mean that most *p*-values were highly statistically significant and instead assessed effect modification by stratifying analysis and visually inspecting of the association estimates.

### 2.5. Sensitivity Analyses

To check if the associations was present in low-level areas, we ran a threshold analysis where we stratified data on NO_2_ levels of 15 μg·m^3^. Since asthma medications are prescribe very frequently to small children we excluded children below the age of two in a sensitivity analysis. In another analysis, we delayed start of follow-up one year, to 1 January 2008. We also restricted the analysis to those children and adolescents who resided in the same address for at least two year from start of follow-up. Finally, we investigated if patterns of missingness among our variables was differential with respect to the outcome or level of exposure. We imputed missing observations in the covariates using a Markov Chain Monte Carlo approach and reran the main analyses to investigate whether our estimates changed. We also calculated a pooled estimate from the county-specific estimates. The main analysis was run also with Cox regression, and mixed logistic regression to take into account the multilevel nature of data (county-level). SAS V.9.2 (SAS, Stockhom, Schweden) software was used to create data sets and run the analyses.

## 3. Results

None of the background factors had a clear univariate association with medications for asthma, except age, with the youngest children as expected being medicated to a higher extent (Table 1). Furthermore, in the univariate analysis there were no marked heterogeneity in NO_2_ with respect to any of the factors in the study (Table 1).

However, in young children (<2 years at study entry), there was an association between smoking during early pregnancy and asthma (data not shown). There was an association between medications for asthma and NO_2_ when adjusted for age with an Odds ratio (OR) of 1.05 (95% Confidence Interval (CI: 1.04–1.06) associated with a 10 µg·m^−3^ increase in NO_2_ (Table 2).

When adjusted for sex, parental education, smoking during pregnancy and BMI in early pregnancy, the OR was attenuated to 1.02 (95% CI: 1.01–1.03; Table 2). The estimate that was pooled from the county-specific estimates was 1.06 (95% CI: 1.02–1.11). The analysis where data was stratified on NO_2_ above and below 15 (µg·m^−3^) indicates that the association between NO_2_ and dispensed asthma medication only seem to be present in areas with higher levels of air pollution, above 15 (µg·m^−3^) in this study (Table 2). When excluding children under the age of two at study entry, the OR in the adjusted analysis was 1.06 (95% CI: 1.04–1.09; Table 2), suggesting that the association between air pollution and asthma medications was stronger after early childhood.

The estimates were not very precise (wide confidence intervals), but there was no clear evidence for medication of psychiatric disorders to influence the association between air pollution and asthma (Table 2). The association between air pollution and asthma medications seemed stronger in children with parents with high education than in children with parents with low education (Table 2). There was weak evidence for levels of socio-economy (education) in the neighborhood to have an impact on the association between NO_2_ and dispensed asthma medication, with an adjusted OR in areas with highest quartile of education level of 1.05 (95% CI: 1.03–1.07). In the first three quartiles, there was no evidence for an association, with ORs close to 1 (Table 2). From Table 1, it is evident that the levels of air pollution were higher in area with the highest quartile of education level, so these results are consistent with the analysis showing an association mainly where NO_2_ concentrations are higher. The dispersion was also highest in quartile 4, with a standard deviation of 11 µg·m^−3^, in quartile 1–3, 6.3, 5.3 and 5.1 µg·m^−3^. The delay of start of follow-up and restricting the analysis to those who resided in the same address at least two years after follow-up did only marginally affect the estimates (data not shown). Adjusting for parental income only marginally affected the estimates (data not shown). There was some evidence of heterogeneity between counties (*p* for effect modification = 0.004), but the results with mixed logistic regression with county as a level was similar to the ordinary logistic regression. The results with Cox regression was similar to the results generated with logistic regression (data not shown).

## 4. Discussion

In this large prospective cohort consisting of more than half of all Swedish children and adolescents, we observed evidence for air pollution to be associated with dispensed medications for asthma, especially in areas with comparatively higher levels of air pollution. We observed no clear evidence for our hypothesis, that stressors linked to low socio-economy or mental health problems would increase susceptibility to the effects of air pollution on the development of asthma On the contrary, the association between air pollution and asthma seemed stronger in children to parents with high education than with low education.

Our hypothesis derived from two earlier studies, where children exposed to a stressful environment at home seemed to have an increased susceptibility for air pollution-related asthma [16,17]. There are several potential explanations for the differences in findings that should be discussed. First of all, the two previous studies were an interview study of 73 children with asthma [16], and a prospective cohort study of 2497 children based on questionnaires [17], whereas we used data from national registers on a cohort of more than half of all Swedish children and adolescents. Using data from national registers has a major advantages in its longitudinal design and the high-quality data, for example selection bias is not a problem. However, the other two studies used more exact measures of stress, namely interviews of life stress [16] and parental stress from a perceived stress scale [17]. We used data on socio-economy and mental health, which although they are very crude indicators of stress, are relevant potential effect modifiers nevertheless. Furthermore, life-style factors may not have been fully accounted for in the analysis since we were restricted to variables that were present in the registers, which could have resulted in bias residual confounding. The potential confounders we had data on did not seem to have strong associations with neither outcome nor exposure however, although the effect estimates were somewhat attenuated when adjusting for them (Table 1 and Table 2). The estimates were quite stable when adjusting for parental income and group-level socio-economy, which may indicate that residual confounding due to socio-economy is not likely. Secondly, another difference compared to the two previous studies was how we defined the outcome; namely as dispensing at least two asthma medications during follow-up, and we did not use information on diagnosis (doctor-diagnosed new onset asthma during 3 years of follow-up in one study [17] and biologic and clinical outcomes in children with asthma in the other study [16]). Medication use to describe health is increasingly used in the Nordic countries, where national registers provide the opportunity to do so [26,36,37]. Socioeconomic status could influence the probability to dispense medication, but that would also be true if using diagnosis as an outcome. It is well known that health care seeking behavior is highly dependent on socioeconomic status, also in Sweden which is one of the most equal countries in the world with a Gini coefficient of around 0.30 [38]. However, health care is free for every child younger than 18 years of age in Sweden, and medications are heavily subsidized example with respect to income differences. Our results may thus not be generalized to, or compare well with, countries with less access to welfare and health care. Thirdly, our study was conducted in Sweden, where air pollution are generally quite low (the two other studies were done in California [17] and Vancouver [16]), and we cannot rule out that mental health or socio-economic status would modify the association between air pollution and asthma in areas with higher levels of air pollution.

In the present study we used modeled levels of NO_2_, a marker of traffic-related air pollution, as exposure measure. Exposure misclassification must be considered in any study on long-term exposure to air pollution. We used exposure data from 2010 and assumed that differences (contrasts) in exposure would be similar back in time over the follow-up period (2005–2010), which is probably a reasonable assumption given the comparable short time period. An obvious source of exposure misclassification however is the fact that ambient exposure was modeled outdoors at the home address and that exposure at school, day-care or indoor exposure were not taken into account. We know from previous work that actual exposure may correlate only mildly with modelled outdoor exposure [39]. Furthermore, we used data on air pollution and start of follow-up as a marker for long-term exposure to air pollution. The validity of that assumption could of course be questioned, but we used a similar approach in the European Study of Cohorts for Air Pollution Effects (ESCAPE), and for that study we showed that when people changed residential address the air pollution concentrations were often similar at the new and old address. Exposure misclassification could theoretically bias the estimates both away and towards the null, if the misclassification is differential with respect to the outcome. Such misclassification is easy to imagine, for example if cohort members with respiratory symptoms and low socio-economy stay indoors more than cohort members with respiratory symptoms and high socio-economy, but it is difficult to speculate in size and direction of bias from such differential exposure misclassification. We have used the same approach as in many other air pollution epidemiology studies where we have been able to observe associations. For example, we observed strong associations between dispensed medications for psychiatric disorders and air pollution using almost exactly the same cohort and exposure measure as in the present study [26]. We believe, therefore, that exposure misclassification is an unlikely explanation for the results of the present study, at least if any true causal effect modification from any of the variables investigated on the association between traffic air pollution and asthma would be strong.

Furthermore, the associations cannot be explained by heterogeneity in prevalence or exposure across Sweden, as the pooled estimate (from county-specific estimates) seemed slightly higher than the non-pooled estimate. It is somewhat surprising that smoking during pregnancy did not seem to be clearly associated with asthma in the children in our study, since smoking during pregnancy seem to be a risk factor for asthma in children and adolescent [40,41], particularly for asthma or asthmatic symptoms during the first years of life [42,43,44]. An association between smoking during pregnancy and asthma in young children is supported in our data, where there was a univariate association between smoking during early pregnancy and the outcome, but only in children which were very young (<2 years at study entry).

## 5. Conclusions

In conclusion, we observed associations between dispensed asthma medications and levels of air pollution at the home address, in areas where levels were comparatively high (NO_2_ annual mean ≥15 (µg·m^−3^). We observed evidence for the association to be stronger in children to parents with high education, but we did not observe support for our hypothesis that stressors linked to socio-economy or mental health problems would increase susceptibility to the effects of air pollution on the development of asthma.

## Figures and Tables

**Table 1:** Descriptive data for variables used in the study, stratified by asthma medication and mean levels of NO_2_ (µg·m^−3^).

Variables	Categories	Asthma Medication *		Mean NO_2_
		No	Yes	(µg·m^−3^)
		N	N (%)	
Boys		566,571	50,809 (8)	10.2
Girls		558,711	39,663 (7)	10.3
Age at baseline	<2	303,033	56,014 (16)	11.4
	2 < 5	134,476	7466 (5)	10.0
	5 < 10	229,546	11,037 (5)	9.7
	10 < 15	272,821	12,576 (4)	9.6
	15–18	185,406	3379 (2)	9.8
Smoking early pregnancy	Missing	184,807	11,722 (6)	11.1
	No	813,138	69,803 (8)	10.1
	Yes <10 cig per day	83,406	6262 (7)	9.9
	Yes ≥10 cig per day	43,931	2685 (6)	9.8
Mother’s education	Missing	95,790	6723 (7)	12.1
	Elementary school	124,997	10,344 (8)	11.1
	Only upper secondary school or Post-secondary education < 2 years	453,873	36,871 (8)	9.4
	Post-secondary education 2 ≤ 4 years	336,162	27,173 (7)	10.3
	Post-secondary education ≥ 4 years	114,550	9361 (8)	11.1
Father’s education	Missing	109,437	7320 (6)	12.0
	Elementary school	148,970	11,815 (7)	10.2
	Only upper secondary school or Post-secondary education < 2 years	477,250	40,059 (8)	9.4
	Post-secondary education 2 ≤ 4 years	258,744	21,194 (8)	10.7
	Post-secondary education ≥ 4 years	130,881	10,084 (7)	11.2
Group level education **	quartile 1 (<14%)	296,380	23,106 (7)	
Group level education **	quartile 2 (14–<20%)	262,997	20,879 (7)	8.9
Group level education **	quartile 3 (20% < 32%)	292,250	24,230 (8)	9.0
Group level education **	quartile 4 (≥32%)	273,655	22,257 (8)	14.2
Mother unemployed at baseline	No	1,033,154	85,288 (7)	10.2
	Yes	92,128	7884 (8)	10.5
Father unemployed at baseline	No	1,067,643	85,544 (7)	10.2
	Yes	57,639	4928 (8)	11.5
Analeptika (N06) at baseline	No	1,115,524	90,028 (7)	10.3
	Yes	9758	444 (4)	9.5
Neuroleptika (N05) at baseline	No	1,117,444	89,894 (7)	10.3
	Yes	7838	578 (7)	10.1
		Mean		Spearman rho
BMI early pregnancy		23.9	24.5	−0.04
Mother’s income baseline (SEK)		162,600	151,200	−0.03
Father’s income baseline (SEK)		280,000	267,800	−0.06
NO_2_ baseline (µg·m^−3^)		10.2	10.5	1

* Defined as dispensing at least two medications with the ATC-codes R03AC, R03AK, R03BA, R03BC, R03CC and R03DC during the study period (2007–2010); ** Proportion in the neighborhood (SAMS area) with three or more years of undergraduate studies in the age category 25–65 years.

**Table 2:** Odds ratios (ORs) and their 95% Confidence Intervals (CIs) for asthma medication in association with a 10 (µg·m^−3^) increase in NO_2_ for all cohort members, and in sub-groups of the cohort.

	OR	95% CI		
	Age-Adjusted		Adjusted ^4^	
All	1.05	1.04–1.06	1.02	1.01–1.03
Sub–groups				
Mother low education ^1^	1.01	0.98–1.05	0.99	(0.95–1.02)
Mother high education ^2^	1.04	1.01–1.06	1.05	1.02–1.09
Low father education ^1^	1.03	0.997–1.06	0.99	0.96–1.027
Father high education ^2^	1.03	1.00–1.05	1.04	1.01–1.07
Unemployment mother baseline	1.03	1.00–1.07	1.00	0.97–1.04
Unemployment father baseline	1.06	1.02–1.10	1.02	0.97–1.06
Age baseline				
<2	1.06	1.04–1.07	1.02	1.01–1.04
2 ≤ 5	1.05	1.01–1.08	1.03	0.99–1.08
5 ≤ 10	1.12	1.08–1.15	1.07	1.03–1.11
10 ≤ 15	1.14	1.11–1.17	1.07	1.03–1.11
15–18	1.10	1.04–1.15	1.00	0.83–1.20
Dispensed neuroleptika (N05) baseline year	1.07	0.95–1.21	1.03	0.87–1.21
Dispensed analeptika (N06) baseline year	1.05	0.92–1.21	1.12	0.90–1.40
Neighborhoodeducation quartile 1 (<14%) ^3^	1.01	0.99–1.03	0.93	0.90–0.96
Neighborhoodl education quartile 2 (14–<20%) ^3^	0.96	0.94–0.99	0.92	(0.89–0.96)
Neighborhood education quartile 3 (20% < 32%) ^3^	1.04	1.01–1.07	1.01	0.98–1.04
Neighborhood education quartile 4 (≥32%) ^3^	1.05	1.03–1.06	1.05	1.03–1.07
Children < 2 years of age	1.06	1.04–1.07	1.02	1.01–1.04
Children ≥ 2 years of age	1.11	1.09–1.13	1.06	1.04–1.09
Stockholm	1.04	1.02–1.06	1.02	1.00–1.04
Skåne	1.09	1.07–1.12	1.07	1.05–1.10
Västra Götaland	1.02	1.01–1.04	0.99	0.98–1.01
Västerbotten	1.04	0.98–1.11	1.02	0.95–1.10
NO_2_ <15 (µg·m^−3^)	0.97	0.95–0.99	0.93	0.91–0.95
NO_2_ ≥15 (µg·m^−3^)	1.11	1.09–1.14	1.09	1.07–1.12

^1^ Elementary school only; ^2^ Post-secondary education ≥ 4 years; ^3^ The proportion of the population in the neighborhood (SAMS-area) with three or more years of undergraduate studies in the age category 25–65 years; ^4^ Adjusted for age at baseline, sex, four categories of parental (maternal and paternal) education and for three categories of smoking and a continuous measure of BMI during early pregnancy.
